# Genome Sequence of *Chrysotila roscoffensis,* a Coccolithphore Contributed to Global Biogeochemical Cycles

**DOI:** 10.3390/genes13010040

**Published:** 2021-12-23

**Authors:** Ran Meng, Lin Zhang, Chengxu Zhou, Kai Liao, Peng Xiao, Qijun Luo, Jilin Xu, Yanze Cui, Xiaodi Hu, Xiaojun Yan

**Affiliations:** 1 College of Food and Pharmaceutical Sciences, Ningbo University, Ningbo 315211, China; mengran@nbu.edu.cn (R.M.); zhouchengxu@nbu.edu.cn (C.Z.); 2 Li Dak Sum Yip Yio Chin Kenneth Li Marine Biopharmaceutical Research Center, Ningbo University, Ningbo 315211, China; 3 School of Marine Science, Ningbo University, Ningbo 315211, China; zhanglin@nbu.edu.cn (L.Z.); liaokai@nbu.edu.cn (K.L.); 18970639403@163.com (P.X.); luoqijun@nbu.edu.cn (Q.L.); xujilin@nbu.edu.cn (J.X.); 4 Novogene Bioinformatics Institute, Beijing 100083, China; cuiyanze@novogene.com; 5 School of Marine Science, Zhejiang Ocean University, Zhoushan 316022, China

**Keywords:** coccolithophores, *Chrysotila roscoffensis*, phenotypic diversification, calcification

## Abstract

*Chrysotila* is a genus of coccolithophores. Together with *Emiliania*, it is one of the representative genera in the Haptophyta which have been extensively studied. They are photosynthetic unicellular marine algae sharing the common characteristic of the production of CaCO_3_ platelets (coccoliths) on the surface of their cells and are crucial contributors to global biogeochemical cycles. Here, we report the genome assembly of *Chrysotila roscoffensis*. The assembled genome size was ~636 Mb distributed across 769 scaffolds with N50 of 1.63 Mb, and maximum contig length of ~2.6 Mb. Repetitive elements accounted for approximately 59% of the genome. A total of 23,341 genes were predicted from *C. roscoffensis* genome. The divergence time between *C. roscoffensis* and *Emiliania huxleyi* was estimated to be around 537.6 Mya. Gene families related to cytoskeleton, cellular motility and morphology, and ion transport were expanded. The genome of *C. roscoffensis* will provide a foundation for understanding the genetic and phenotypic diversification and calcification mechanisms of coccolithophores.

## 1. Introduction

Coccolithophores, belonging to the Haptophyta, are photosynthetic unicellular marine algae sharing the common characteristic of the production of CaCO_3_ platelets (coccoliths) on the surface of their cells. They are globally distributed across all oceans except the polar ones, with some species forming blooms that can be observed from artificial satellites [1]. Coccolithophores play a fundamental role in the marine carbon cycle through the fixation of inorganic carbon by photosynthesis (the organic carbon pump) and the export of CO_2_ during calcification (the carbonate counter pump) [2]. Consequentially, they are thought to be responsible for about 10% of global carbon fixation [3] and to produce up to 50% of oceanic CaCO_3_ [4]. Coccolithophores also affect the global sulfur cycle through their production of dimethylsulfoniopropionate (DMSP), the major precursor of atmospheric dimethyl sulfide (DMS) [5]. In addition, coccoliths provide ballast that drives the transfer of particulate organic matter to the deep ocean [6]. 

Given the ecological and biogeochemical importance of coccolith formation, the mechanisms of calcification have raised a considerable interest and research. The calcification is common for a number of organisms, including unicellular organisms, invertebrates, and vertebrates, but it has unique cellular and biochemical characteristics in the coccolithophores. Firstly, the sites of calcification in the coccolithophores differ from those in other organisms. These sites are either extracellular or intercellular for most biological calcification, while coccoliths are produced intracellularly in a Golgi-derived coccolith vesicle (CV) [7]. Secondly, the composition of the organic matrix is different between the coccolithophores and other organisms. Acidic polysaccharides, in contrast to proteins found in other organisms, such as bivalve mollusks [8], crayfish [9], pearl oyster [10], and fishotolith [11], are the main component of the organic matrix and are predominantly associated with coccolith formation in the coccolithophores [12]. Until now, the molecular mechanisms and regulators underlying characteristics of calcification in the coccolithophores are still not fully elucidated. 

There are approximately 200 extant species of coccolithophores [13]; *Emiliania* and *Chrysotila* (formerly *Pleurochrysis*) are the two most explored genera. To our knowledge, only *Emiliania huxleyi* genome is available in the coccolithophores, even in the Haptophyta [14]. Moreover, these two genera exhibit high degree of genetic and phenotypic variations. For example, the gene content varied from 10% to 30% among *E. huxleyi* strains [15]. *C. carterae* (formerly *Pleurochrysis carterae*) calcification takes place at night, whereas *E. huxleyi* coccolith is mainly formed during day [16]. In *Emiliania*, the reticular body (RB) is closely connected to CV and is important in providing raw material for calcification [17] but appears to be absent in *Chrysotila* [7]. Three types of acidic polysaccharides (PS1, PS2, and PS3) were identified in *Chrysotila*, but *Emiliania* lacks PS1 and PS2, which deliver Ca^2+^ to CV in *Chrysotila* [12]. There are very limited data on the evolution and mechanisms of these variability in the coccolithophores. 

While *Emiliania* species distribute globally in almost all ocean ecosystems, species of genus *Chrysotila* was mainly found in coastal, estuarine, brackish waters and in marine aquaculture pools. Notorious foaming blooms of *Chrysotila* species frequently occur in these areas. Some species in genus *Chrysotila* were lethal to brine shrimp [18], a model organism in many toxicological research. However, the mechanism of the lethal effects is not unraveled. Non-calcified filamentous colonies in the life cycle of *Chrysotila* species is typical heteromorphic characteristic in this genus [19]. In the present study, we report on the assembly and annotation of the *C. roscoffensis* genome. The data will provide a foundation for understanding the genetic and phenotypic diversification and calcification mechanisms of coccolithophore, a key player in the global biogeochemical cycles. 

## 2. Materials and Methods 

### 2.1. C. roscoffensis Strain and DNA Extraction

**Figure 1:**
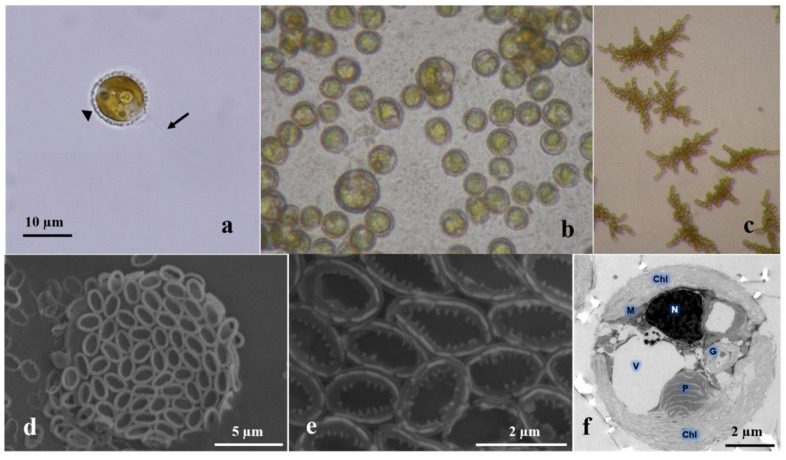
Microscopic images of *Chrysotila roscoffensis* (strain NMBjih026-8). (**a**) motile coccolith-bearing cell, showing two flagellates (arrow) and coccolith (arrow head). (**b**) nonmotile coccolith-bearing cells. (**c**) non-calcified filamentous colonies. (**d**) scanning electron microscope (SEM) image of coccolith-bearing cell. (**e**) SEM image of coccoliths. (**f**) transmission electron microscope (TEM) image of coccolith-bearing cell. Chl: chloroplast; G: Golgi apparatus; M: mitochondrion; N: nucleus; P: pyrenoid; and V: vacuole.

Genomic DNA from *C. roscoffensis* (strain NMBjih026-8, Figure 1) was used for library construction and sequencing. The strain was originally isolated from coastal waters in Xiangshan Bay (N 29°32′48.44″, E 121°48′34.62″), near Ningbo, Zhejiang, China, in January 2009. A unialgal culture was established and kept in the Microalgae Collection Center of Ningbo University, Zhejiang, China. 

For DNA isolation, fresh culture of motile coccolith-bearing cells was inoculated and grown in sterilized natural seawater (pH 8.30, salinity 24‰) enriched with f/2-Si culture medium, at 20 °C in light density of 60 µmol/(m^2^∙s) with a light/dark cycle of 12 h:12 h. To minimize bacterial contamination, the culture medium was supplemented with the appropriate antibiotics: 100 mg/L ampicillin, 100 mg/L kanamycin, 100 mg/L neomycin, 100 mg/L streptomycin, and 30 mg/L chloramphenicol. The algal cells were harvested at exponential phase, and total genomic DNA was extracted with the Plant DNA Kit (Tiangen, Beijing, China) under the guidance of the manufacturer’s instructions. One percent of agarose gel electrophoresis and Qubit Fluorometer were used to check the quality and quantity of the isolated DNA, ensuring in the final concentration ≥ 20 ng/uL, a total amount ≥ 50 ng, no or slightly degraded, and main DNA band ≥ 5 Kb. 

### 2.2. Library Construction and Sequencing

Three libraries were constructed and sequenced. A short DNA library with an insert size of 350 bp was prepared and sequenced on Illumina Xten platform as 150 bp paired-end reads. One SMRT Bell library with an insert size of 20 kb was constructed, and the sequencing was performed on PacBio Sequel platform. The linked read sequencing library was also performed on a 10X Genomics GemCode platform. 

### 2.3. Genome Size Estimation and De Novo Genome Assembly

Genome size and heterozygosity of *C. roscoffensis* were estimated by setting k-mer to 17. The 17-mer frequency distribution analysis of all clean reads from the Illumina platform was performed using SOAPdenovo [20]. PacBio reads were subjected to de novo assembly using FALCON (https://github.com/PacificBiosciences/FALCON/, accessed on 19 December 2021). First, error-correcting PacBio raw sequencing data was performed using FALCON. After correction, all reads were aligned to each other and assembled into contigs and these contig sequences were polished using Quiver algorithm. The draft assembly was corrected with Pilon [21] based on the 52.41× high-quality Illumina sequencing reads to collect enough corrected genome sequences. After that, the 10× Genomics data was aligned to the assembly by BWA [22] using default settings and the quality of assembly was assessed by mapping the clean short insert size reads to the scaffolds. Finally, we also evaluated the level of genome completeness of the final genome assembly using CEGMA [23].

### 2.4. Repetitive Sequences Annotation

Repeat sequences were identified and classified using a combination of de novo and homology-based approaches. The *ab initio* prediction program RepeatModeler (http://www.repeatmasker.org/RepeatModeler.html) was employed to construct a de novo repeat library from the *C. roscoffensis* genomes. The homology-based annotation was performed by mapping the *C. roscoffensis* genomes onto Repbase database (http://www.girinst.org/) and TE protein database using RepeatMasker (http://www.repeatmasker.org/RMDownload.html) and RepeatProteinMask software [24], respectively. Tandem repeats were identified using Tandem Repeats Finder [25]. 

### 2.5. Genome Annotation 

Homolog-based, de novo, and transcriptome-based methods were used to construct the gene model set. Homolog proteins sequences of *E. huxleyi*, *Phaeodactylum tricornutum*, *Chlamydomonas reinhardtii*, *Chlorella variabilis*, *Arabidopsis thaliana*, and *Volvox carteri* were downloaded from Ensemble (http://plants.ensembl.org/index.html) and NCBI (https://www.ncbi.nlm.nih.gov/). The gene models were extracted using GeneWise [26] in accordance with the alignments of the homolog proteins sequences to the repeat-masked genomes. We adopted five ab initio gene-prediction software: Augustus (version 2.5.5) [27], Genscan (version 1.0) [28], GlimmerHMM (version 3.0.1) [29], Geneid [30], and SNAP [31] to perform the de novo gene models predictions. RNA-seq data were mapped to the repeat-masked genomes using Tophat (version 2.0.8) [32], and Cufflinks (version 2.1.1) [33] (http://cufflinks.cbcb.umd.edu/). In addition, we de novo assembled RNA-seq data into several pseudo-ESTs by Trinity [34]. These pseudo-ESTs were also aligned to the repeat-masked genomes and gene models were predicted by PASA [35]. The EvidenceModeler (EVM) [36] was adopted to combine all of the Homo-set, Cufflinks-set, PASA-T-set and ab initio gene sets to generate a consensus and non-redundant reference gene set. 

We annotated the gene functions according to the alignments to two integrated protein sequence databases (SwissProt and NR) by BLASTP with an e-value cutoff of at 1e^−5^. The InterProScan [37] was adopted to search motifs and conserved functional domains using Pfam and GO databases. The pathways involved in interactions, reactions, and relationships among genes were assigned by BLAST searching the KEGG databases [38], with an E-value cutoff at 1e^−5^. 

### 2.6. Phylogenetic and Comparative Genomic Analysis

We performed comparative analysis between the *C. roscoffensis* genes and the genes identified from *C. reinhardtii*, *C. eustigma*, *Chromochloris zofingiensis*, *Micromonas pusilla*, *Chlorella sorokiniana*, *Chara braunii*, *Thalassiosira oceanica*, *Thalassiosira pseudonana*, *P. tricornutum*, *Aureococcus anophagefferens*, *Saccharina japonica*, *E. huxleyi*, *Symbiodinium microadriaticum*, *Porphyra umbilicalis*, *Galdieria sulphuraria*, *Chondrus crispus*, *Bigelowiella natans*, *A. thaliana* and *Oryza sativa* (Table S1). The genes of each species were filtered as follows: first, only the longest transcript was retained when multiple transcripts are present in one gene; second, only the genes with an encoding length longer than 50 amino acids were retained. Then, the similarity of protein sequences between pairs of all species was obtained by blastp with the e-value 1e^−5^. OrthoMCL (http://orthomcl.org/orthomcl/) [39] was applied to cluster into paralogous and orthologous among 20 species protein datasets with the inflation parameter 1.5. MUSCLE [40] (http://www.drive5.com/muscle/)was adopted to align the protein sequences of each of 25 one-to-one single-copy gene families shared by all species, and all the results were combined into a super alignment matrix. Then, the 20-species phylogenetic tree was constructed using RaxML [41] (http://sco.h-its.org/exelixis/web/software/raxml/index.html) with the maximum likelihood method, and the bootstrap was 100. B. natans was selected as the outgroup. We performed divergence dating based on the phylogenetic analysis using MCMCtree in PAML package [42,43]. 

The gene families that expanded and contracted in all genomes were identified using CAFÉ [44] based on phylogenetic analysis. To further functionally annotate the expanded gene families, the gene ontology (GO) term was retrieved from InterProScan results and the enrichment analysis was performed.

## 3. Results and Discussion

### 3.1. Genome Analysis of C. roscoffensis

**Figure 2:**
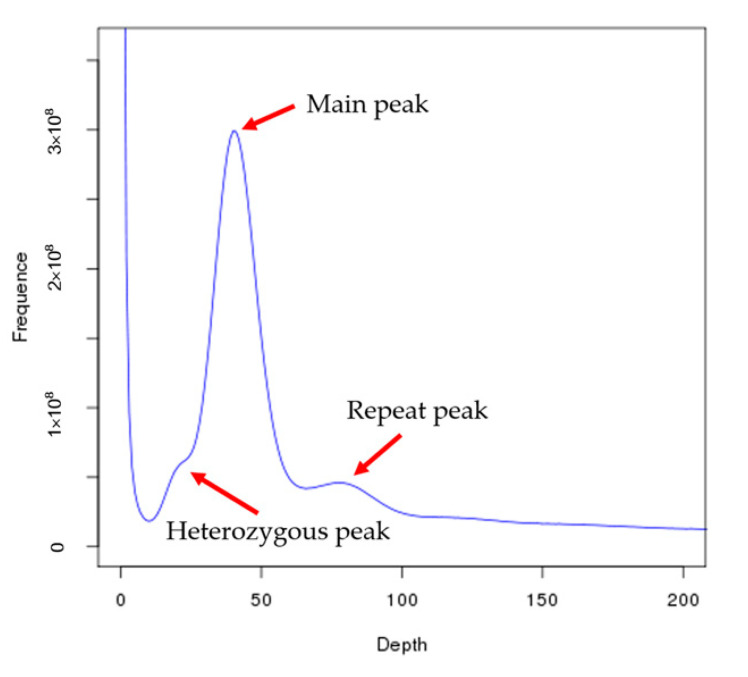
17 K-mer analysis for estimating the genome size of *C. roscoffensis.* The distribution of 17-mer was calculated using jellyfish (version2.1.3) based on the sequencing data from short insert size libraries and the genome size was estimated based on the formula: genome size = total_kmer_num / kmer_depth, where total_kmer_num is the total number of K-mer and kmer_depth indicates the peak position on the K-mer frequency distribution map. Heterozygous peak indicates the genome heterozygosity, repeat peak represents the repeat rate of the genome.

Based on the total number of k-mers (26,900,644,184), the *C. roscoffensis* genome size was calculated to be approximately 674.07 Mb and the heterozygosity was 0.64%, which indicated a relatively lower intraspecific variation compared to *E. huxleyi* [14] (Figure 2 and Table 1). To prepare for following de novo assembly, we filtered the low quality, duplicated, and adapter-containing reads generated by Illumina Xten platform to ensure high accuracy. After that, a total of 35.33 Gb (52.41-fold coverage of the genome) data were retained (Table 2). A total of 53.12 Gb (78.80-fold coverage of the genome) PacBio sequencing data were produced for the assembly (Table 2). The 93.22 Gb library was sequenced with 150 bp paired-end reads were generated by an Illumina HiSeq X Ten platform (Table 2). The assembled genome size was ~636 Mb distributed across 769 scaffolds (Table 3). The final assembly result is close to the estimated genome size based on 17-mer analyses. Almost 85.30% of reads could successfully align to final assembly (Table S2). CEGMA analysis showed that 81.05% conserved core eukaryotic genes could be captured in our genome, of which 75.00% were complete (Table S3). These results indicated that the genome assembled in this paper contained comprehensive genomic information. 

### 3.2. Genome Annotation 

The results show that 58.54% of *C. roscoffensis* genome consists of repetitive elements (Table 4). Among these repeats, 53.67% could be divided into known repeat families. Long-terminal repeats (LTRs) were the most abundant repeat family, accounting for 37.04% of the genome size (Table 5). The second largest family in *C. roscoffensis* was DNA elements, which account for 5.66% of the genome size. A total of 23,341 genes were yielded from *C. roscoffensis* genome and the average lengths of CDS, exon, and intron were 1596 bp, 277 bp, and 719 bp, respectively (Table 6). Finally, a total of 23,216 genes were predicted to be functional, accounting for 99.5% of all genes in *C. roscoffensis* genome (Table 7).

### 3.3. Phylogenetic and Comparative Genomic Analysis 

**Figure 3:**
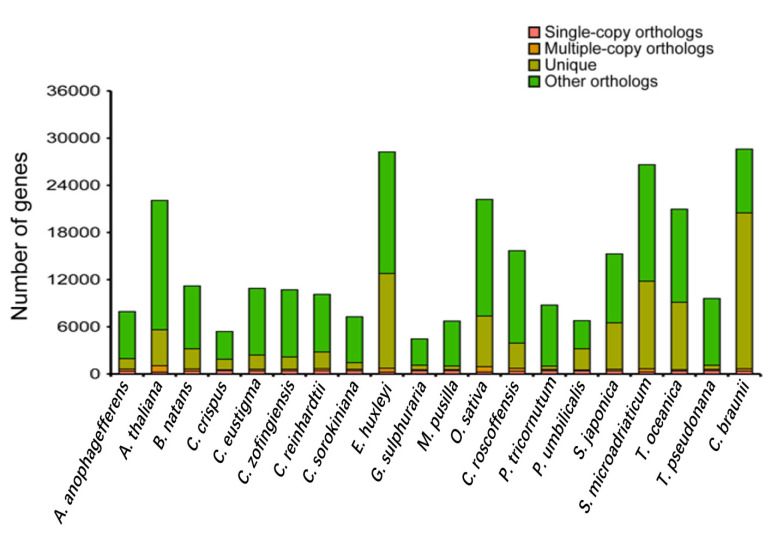
The distribution of genes in *Aureococcus anophagefferens*, *Arabidopsis thaliana*, *Bigelowiella natans*, *Chondrus crispus*, *Chlamydomonas eustigma*, *Chromochloris zofingiensis*, *Chlamydomonas reinhardtii*, *Chlorella sorokiniana*, *Emiliania huxleyi*, *Galdieria sulphuraria*, *Micromonas pusilla*, *Oryza sativa*, *Chrysotila roscoffensis*, *Phaeodactylum tricornutum*, *Porphyra umbilicalis*, *Saccharina japonica*, *Symbiodinium microadriaticum*, *Thalassiosira oceanica*, *Thalassiosira pseudonana* and *Chara braunii*.

**Figure 4:**
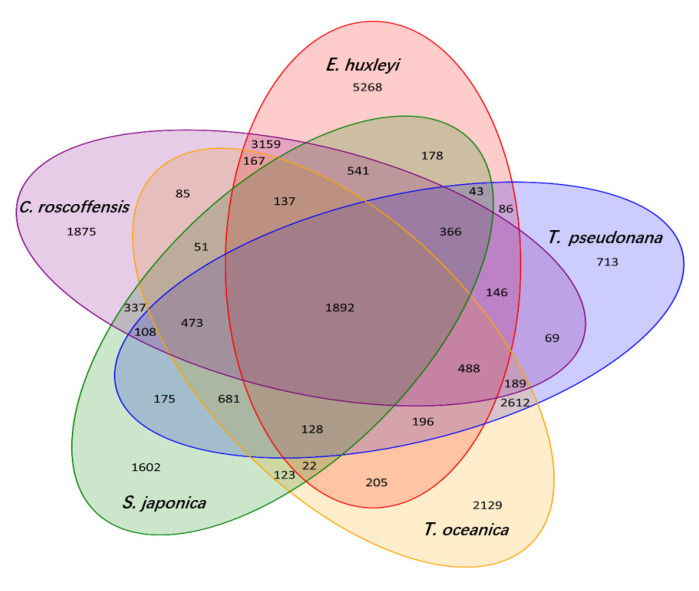
Common and unique gene families in five groups. Venn diagram showing comparison of shared and unique protein-coding genes among *Chrysotila roscoffensis*, *Emiliania huxleyi*, *Thalassiosira pseudonana, Thalassiosira oceanica*, and *Saccharina japonica* based on orthology analysis.

The distribution of genes in *C. roscoffensis* and other 19 species was shown in Figure 3. Additionally, common and unique gene families in *C. roscoffensis*, *E. huxleyi*, *S. japonica*, *T. oceanica*, and *T. pseudonana* were presented in Figure 4. Phylogenetic analysis has shown that the divergence time between *C. roscoffensis* and *E. huxleyi* is estimated to be around 537.6 Mya (Figure S1). This result suggested the divergence between *C. roscoffensis* and *E. huxleyi* was much earlier than previously predicted (approximately 250 Mya) [45].

### 3.4. Expanded Coccoliths-Related Gene Families 

Compared with *E. huxleyi*, there were 22 significantly expanded gene families and 39 significantly contracted gene families were identified in *C. roscoffensis* (Figure S1). There are 60 GO terms were significantly enriched among the expanded gene families (*p* ≤ 0.05, Table S4). Among these significantly enriched GO terms, there are 16 terms associated with cytoskeleton, cellular motility and morphology, such as ‘dynein complex’, ‘cellular component movement’, ‘microtubule motor activity’, ‘microtubule-based movement’, ‘microtubule-based process’, ‘motor activity’, ‘microtubule cytoskeleton’, ‘microtubule associated complex’, ‘cytoskeletal part’, ‘cytoskeleton’, ‘anatomical structure morphogenesis’, ‘cilium or flagellum-dependent cell motility’, ‘axonemal dynein complex’, ‘cell morphogenesis’, ‘anatomical structure development’, and ‘non-membrane-bounded organelle’. The cytoskeleton plays fundamental roles in intracellular transport, secretion of cell wall materials, and the regulation of cell morphology in many eukaryotes [46]. In several species, the disruption of cytoskeleton prevents the secretion of coccoliths, resulting in the formation of malformed coccoliths [47,48]. The roles of cytoskeleton in calcification, such as regulating the shape of the coccolith vesicle and controlling vesicle and cell movements by interacting with the membrane trafficking system, have been proposed [5]. Thus, the significant expansion of families of genes associated with cytoskeleton in *C. roscoffensis* leads to a hypothesis that the calcification and morphological characteristics are associated with cytoskeleton and cellular motility. 

Here, we also identified a set of significantly enriched GO terms associated with ion transport. The coccolith is produced in a Golgi-derived CV and then is secreted to the cell surface through exocytotic pathways [5]. The calcification process presents a remarkable case of transport physiology, requiring rapid rates uptake of Ca^2+^ and HCO_3_^−^ from the surrounding seawater into the CV and meanwhile removal of the produced H^+^ which may exert pressure on the internal pH homeostasis of the cell [49,50]. The expansion of ion transport process related genes could reflect the demand for delivery of substrates and removal of products during calcification in *C. roscoffensis*. 

## 4. Conclusions

In conclusion, we report the genome sequencing, assembly, and annotation of the coccolithophore, *C. roscoffensis*. The assembled genome size was ~636 Mb distributed across 769 scaffolds with N50 of 1.63 Mb, and maximum contig length of ~2.6 Mb. Repetitive elements accounted for approximately 59% of the genome. A total of 23,341 genes were predicted from *C. roscoffensis* genome. The divergence time between *C. roscoffensis* and *E. huxleyi* was estimated to be around 537.6 Mya. Gene families related to cytoskeleton, cellular motility, and morphology and ion transport were expanded. These data are valuable genetic resource for elucidating coccolithophore biology. 

## Figures and Tables

**Table 1:** Survey statistic results of *C. roscoffensis*.

Species	Total Base (Gb)	K-Mer	K-Mer Number	K-Mer Depth	Genome Size (Mb)	Revised Genome Size (Mb)	Heterozygous Ratio (%)	Repeat Ratio (%)
*C. roscoffensis*	34.24	17	26,900,644,184	39	689.76	674.07	0.64	69.45

**Table 2:** Sequencing data statistics of *C. roscoffensis*.

Pair-End Libraries	Insert Size	Total Data (G)	Read Length (bp)	Sequence Coverage (X)
Illumina reads	350 bp	35.33	150	52.41
Pacbio reads		53.12		78.80
10X Genomics		93.22	150	138.29
Total		181.67		269.51

**Table 3:** Summary of the final genome assembly of *C. roscoffensis*.

Sample ID	Length		Number	
	Contig ** (bp)	Scaffold (bp)	Contig **	Scaffold
Total	629,886,791	635,699,922	2167	769
Max	2,590,224	12,677,996		
Number ≥ 2000			2167	769
N50	441,430	1,631,423	434	111
N60	354,170	1,228,002	593	156
N70	281,606	954,517	791	215
N80	208,186	651,419	1053	296
N90	141,820	391,115	1414	420

** Contig after scaffolding.

**Table 4:** Summary of repeat contents in *C. roscoffensis* genome.

Type	Repeat Size	% of Genome
Trf	74,813,341	11.493833
Repeatmasker	327,015,645	50.240549
Proteinmask	67,002,054	10.293758
Total	381,019,300	58.537318

**Table 5:** Statistics of transposable element (TE) classification in *C. roscoffensis* genome.

	Denovo + Repbase		TE Proteins		Combined TEs (All without Trf)	
	Length (bp)	% in Genome	Length (bp)	% in Genome	Length (bp)	% in Genome
DNA	33,824,343	5.196551	4,008,971	0.615912	36,809,695	5.655201
LINE	7,142,576	1.097339	2,411,479	0.370484	8,374,515	1.286606
SINE	196,696	0.030219	0	0	196,696	0.030219
LTR	236,201,808	36.288504	60,676,043	9.321871	241,112,694	37.042981
Other	0	0	0	0	0	0
Satellite	3,083,747	0.473767	0	0	3,083,747	0.473767
Simple_repeat	25,608,316	3.934295	0	0	25,608,316	3.934295
Unknown	31,651,266	4.862694	0	0	31,651,266	4.862694
Total	327,015,645	50.240549	67,002,054	10.293758	331,759,778	50.969407

**Table 6:** Basic statistical results of gene structure prediction of *C. roscoffensis* genome.

Gene Set		Number	Average Gene Length (bp)	Average CDS Length (bp)	Average Exons Per Gene	Average Exon Length (bp)	Average Intron Length (bp)
*De novo*	Augustus	43,490	3611.96	1504.35	4.12	365.32	675.96
	GlimmerHMM	313,490	1985.29	1123.67	3.85	292.11	302.67
	SNAP	102,913	1468.91	842.4	2.1	401.8	571.35
	Geneid	104,522	2507.4	1130.75	2.74	412.95	791.97
	Genscan	55,474	8837.72	2586.56	8.02	322.45	890.29
Homolog	*Emiliania huxleyi*	21,246	1339.63	695.35	1.75	397.92	861.93
	*Phaeodactylum tricornutum*	5755	1577.79	782.52	2.07	377.49	741.18
	*Chlamydomonas reinhardtii*	12,700	1608.62	938.93	1.92	489.67	729.92
	*Chlorella variabilis*	5117	1463.96	732.1	1.98	369.66	746.45
	*Volvox carteri*	13,333	922.29	609.61	1.47	413.34	658.52
	*Arabidopsis thaliana*	13,684	1312.01	892.26	1.47	609.02	902.56
RNA-seq	Cufflinks	43,799	7548.43	2585.25	6.42	402.61	915.52
	PASA	76,439	3568.24	1093.39	4.32	253.27	746.1
EVM		47,323	3839.76	1523.32	4.34	351	693.55
PASA-update		46,875	3848.09	1550.63	4.33	357.92	689.43
Final set		23,341	5013.31	1596.61	5.75	277.68	719.32

**Table 7:** The statistical results of gene function annotation of *C. roscoffensis* genome.

Database		Annotated Num	Annotated Percent (%)
**NR**		16,841	72.2
Swiss-Prot		11,919	51.1
KEGG		11,807	50.6
InterPro	All	23,179	99.3
	Pfam	12,799	54.8
	GO	21,194	90.8
Annotated		23,216	99.5
Total		23,341	-

## Data Availability

The raw sequencing data of the genomic and the transcriptome are available via NCBI with the BioProject accession number PRJNA648277 and BioSample accession number SAMN15644355. The assembly data have been deposited in NCBI under project accession No. SAMN15637150.

## Supplementary Materials

The following are available online at https://www.mdpi.com/article/10.3390/genes13010040/s1, Figure S1: Estimation of divergence time and expansion and contraction gene families in *C. roscoffensis*, Table S1: Basic statistical results of *C. roscoffensis* and relative species, Table S2. Coverage statistics of *C. roscoffensis* genome, Table S3. Assessment the gene coverage rate using CEGMA and Table S4. Enriched GO terms of expanded genes in *C. roscoffensis* genome assembly.
