# Supplementary material for: Genome Sequence of Chrysotila roscoffensis, a Coccolithphore Contributed to Global Biogeochemical Cycles
Source: Genes (Basel). 2021 Dec 23;13(1):40. doi: 10.3390/genes13010040 (PMC8775090; doi:10.3390/genes13010040)
Supplement: Supplementary file 1 [file genes-13-00040-s001.zip › genes-1443133-supplementary.pdf]

Table S1. Basic statistical results of *C. roscoffensis* and relative species.

Species	Name	#gene
Cros	*Chrysotila roscoffensis*	23,341
Crei	*Chlamydomonas reinhardtii*	14,400
Ceus	*Chlamydomonas eustigma*	14,062
Czof	*Chromochloris zofingiensis*	15,330
Mpus	*Micromonas pusilla*	10,624
Csor	*Chlorella sorokiniana*	9,526
Cbra	*Chara braunii*	33,791
Toce	*Thalassiosira oceanica*	28,998
Tpse	*Thalassiosira pseudonana*	11,670
Ptri	*Phaeodactylum tricornutum*	10,391
Aano	*Aureococcus anophagefferens*	11,191
Sjap	*Saccharina japonica*	22,662
Ehux	*Emiliania huxleyi*	36,792
Smic	*Symbiodinium microadriaticum*	41,117
Pumb	*Porphyra umbilicalis*	12,979
Gsul	*Galdieria sulphuraria*	6,455
Ccri	*Chondrus crispus*	9,142
Bnat	*Bigelowiella natans*	21,708
Arth	*Arabidopsis thaliana*	26,869
Osat	*Oryza sativa*	34,227

Table S2. Coverage statistics of *C. roscoffensis* genome.

		Percentage
Reads	Mapping rate (%)	85.30
Genome	Average sequencing depth	41.21
	Coverage (%)	96.55
	Coverage at least 4X (%)	95.70
	Coverage at least 10X (%)	94.59
	Coverage at least 20X (%)	89.17

Average sequence depth: The average depth of each base on the genome that is covered by reads; Coverage**:** The proportion of genomes that were covered by reads.

Table S3. Assessment the gene coverage rate using CEGMA.

species	complete		complete + partial	
	**# Prots**	**%completeness**	**# Prots**	**%completeness**
C. roscoffensis	186	75.00	201	81.05

Table S4. Enriched GO terms of expanded genes in *C. roscoffensis* genome assembly.

GO_ID	GO Term	GO Class	P-value	Gene number
GO:0030286	dynein complex	CC	4.04E-30	21
GO:0006928	cellular component movement	BP	1.71E-25	32
GO:0003777	microtubule motor activity	MF	5.48E-22	28
GO:0007018	microtubule-based movement	BP	2.78E-21	27
GO:0007017	microtubule-based process	BP	4.97E-20	30
GO:0005315	inorganic phosphate transmembrane transporter activity	MF	7.06E-19	12
GO:0006817	phosphate ion transport	BP	7.06E-19	12
GO:0003774	motor activity	MF	5.21E-17	30
GO:0015630	microtubule cytoskeleton	CC	3.09E-13	28
GO:0008236	serine-type peptidase activity	MF	8.93E-13	23
GO:0005875	microtubule associated complex	CC	3.13E-12	24
GO:0044430	cytoskeletal part	CC	1.51E-11	29
GO:0004252	serine-type endopeptidase activity	MF	6.60E-11	21
GO:0006508	proteolysis	BP	1.24E-09	35
GO:1902494	catalytic complex	CC	9.34E-09	26
GO:0015698	inorganic anion transport	BP	1.83E-08	18
GO:0005856	cytoskeleton	CC	1.05E-07	34
GO:0004175	endopeptidase activity	MF	1.12E-07	23
GO:0006820	anion transport	BP	3.57E-07	29
GO:0070011	peptidase activity, acting on L-amino acid peptides	MF	1.14E-05	29
GO:0006511	ubiquitin-dependent protein catabolic process	BP	0.000181	8
GO:0015291	secondary active transmembrane transporter activity	MF	0.000182	28
GO:0044422	organelle part	CC	0.001572	44
GO:0009653	anatomical structure morphogenesis	BP	0.002281	4
GO:0008762	UDP-N-acetylmuramate dehydrogenase activity	MF	0.003069	3
GO:0044446	intracellular organelle part	CC	0.004246	37
GO:0000122	negative regulation of transcription from RNA polymerase II promoter	BP	0.005019	2
GO:0004861	cyclin-dependent protein serine/threonine kinase inhibitor activity	MF	0.005019	2
GO:0007601	visual perception	BP	0.006562	5
GO:0022804	active transmembrane transporter activity	MF	0.009135	29
GO:0016525	negative regulation of angiogenesis	BP	0.009139	2
GO:0017111	nucleoside-triphosphatase activity	MF	0.010539	39
GO:0009583	detection of light stimulus	BP	0.01157	4
GO:0043408	regulation of MAPK cascade	BP	0.011605	2
GO:0017069	snRNA binding	MF	0.011605	2
GO:0004415	hyalurononglucosaminidase activity	MF	0.014327	2
GO:0001539	cilium or flagellum-dependent cell motility	BP	0.017289	8
GO:0016495	C-X3-C chemokine receptor activity	MF	0.017296	2
GO:0008792	arginine decarboxylase activity	MF	0.017296	2
GO:0005057	receptor signaling protein activity	MF	0.017668	6
GO:0005858	axonemal dynein complex	CC	0.018779	1
GO:0005102	receptor binding	MF	0.019021	28
GO:0016715	oxidoreductase activity, acting on paired donors, with incorporation or reduction of molecular oxygen, reduced ascorbate as one donor, and incorporation of one atom of oxygen	MF	0.0205	2
GO:0016787	hydrolase activity	MF	0.021548	79
GO:0009416	response to light stimulus	BP	0.023168	6
GO:0000902	cell morphogenesis	BP	0.02393	2
GO:0008295	spermidine biosynthetic process	BP	0.02393	2
GO:0000156	phosphorelay response regulator activity	MF	0.026194	4
GO:0048856	anatomical structure development	BP	0.027364	7
GO:0006527	arginine catabolic process	BP	0.027577	2
GO:0007602	phototransduction	BP	0.0276	3
GO:0031628	opioid receptor binding	MF	0.037206	1
GO:2000479	regulation of cAMP-dependent protein kinase activity	BP	0.037206	1
GO:0004336	galactosylceramidase activity	MF	0.037206	1
GO:0006683	galactosylceramide catabolic process	BP	0.037206	1

GO:0043228	non-membrane-bounded organelle	CC	0.041381	52
GO:0000160	phosphorelay signal transduction system	BP	0.048504	4
GO:0004943	C3a anaphylatoxin receptor activity	MF	0.048739	2
GO:0005118	sevenless binding	MF	0.048739	2

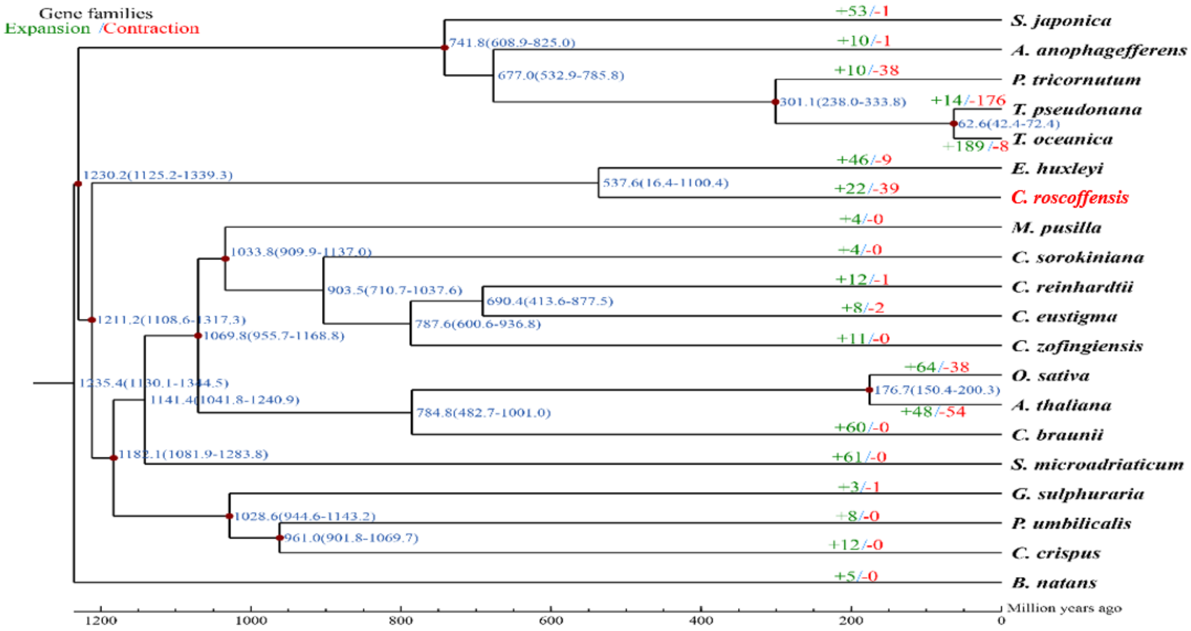

Figure S1. Estimation of divergence time and expansion and contraction gene families in *C. roscoffensis*.
